# A Case of Takotsubo Cardiomyopathy Triggered by Diabetic Ketoacidosis and Hypothermia

**DOI:** 10.7759/cureus.10842

**Published:** 2020-10-07

**Authors:** Mohammed Mhanna, Azizullah Beran, Omar Srour, Sami Ghazaleh, Ahmed Elzanaty

**Affiliations:** 1 Internal Medicine, The University of Toledo, Toledo, USA

**Keywords:** takotsubo cardiomyopathy, stress-induced cardiomyopathy, diabetic ketoacidosis, dka, hypothermia

## Abstract

Takotsubo cardiomyopathy (TC), also recognized as stress-induced cardiomyopathy, is a transient condition of left ventricular (LV) dysfunction, which presents similarly to acute coronary syndrome (ACS) but with normal coronaries. Physical or emotional stressors usually precipitate TC. It is typically a benign condition, with a complete resolution once the triggering cause resolves. There have been a few cases of TC induced by diabetic ketoacidosis (DKA) that have been reported in the literature.

A 50-year-old Caucasian female patient presented with lethargy, in addition to hypothermia and hypotension. Further investigation showed hyperglycemia with metabolic acidosis and ketonemia. Eventually, she was diagnosed with diabetic ketoacidosis (DKA). On Day 2 of the admission, the patient’s condition further deteriorated despite appropriate treatment of DKA. An electrocardiogram (EKG) showed ST-segment elevation in inferior leads, and troponin levels were elevated. Cardiac catheterization showed non-obstructive coronary arteries but a severely reduced cardiac index. Echocardiography showed an ejection fraction (EF) of 25% with global hypokinetic LV. Eventually, the patient was diagnosed with TC or stress-induced cardiomyopathy.

TC should always be suspected in any patient presenting with acute heart failure during DKA treatment. TC is a transient syndrome; however, it can result in dreadful complications, including cardiogenic shock, arrhythmias, or thromboembolic events. Early recognition and timely treatment are pivotal in such cases.

## Introduction

Takotsubo cardiomyopathy (TC) is a transient left ventricular (LV) dysfunction, which was first described in 1990 by Sato et al. in Japan [1]. It is also recognized as stress-induced cardiomyopathy [2]. TC is thought to be underdiagnosed, with the incidence slowly increasing (15-30 patients per 100,000) [3]. At the same time, the prevalence of TC is expected to be 1%-2% of suspected cases with acute coronary syndrome (ACS) [4-6]. TC is triggered by various emotional and physical stress, including acute critical illness, central nervous system disorders, sepsis, and pulmonary diseases [3-5]. Although TC-related acute ventricular dysfunction subsides within a few weeks, it can lead to considerable complications, including arrhythmias, cardiogenic shock, or thromboembolic events [7]. We report a case of stress-induced cardiomyopathy triggered by both DKA and hypothermia.

## Case presentation

A 50-year-old Caucasian woman with a past medical history of diabetes mellitus type 1 was brought to the hospital with lethargy and low blood pressure. Upon arrival to the emergency department (ED), her vital signs were heart rate 96 bpm, respiratory rate 23 breaths/min, blood pressure 74/64 mmHg, and body temperature 90°F. Arterial blood gas showed severe metabolic acidosis (pH: 6.8, HCO3: 2.7 mmol/L, PCO2: 15.4 mmHg, and anion gap: 41 mmol/L). Heart sounds were normal, with no murmurs. Initial laboratory tests revealed creatinine: 4.59 mg/dL, blood glucose: 1637 mg/dL, potassium: 7.6 mmol/L, and beta-hydroxybutyrate 14.33 mmol/L. Urinalysis showed significant glucosuria and ketonuria. Computed tomography (CT) of the brain and chest X-ray (CXR) were unremarkable. She was transferred to the intensive care unit (ICU) for further management. The patient received empiric antibiotics, and she was started on the DKA protocol with intravenous (IV) fluids and insulin drip. The patient's altered mental status continued to deteriorate with a Glasgow Coma Scale score of 8/15 (E2V2M4), and she was subsequently intubated and mechanically ventilated for airway protection.

On admission, the patient's initial troponin levels were mildly elevated at 0.21 ng/ml, and initial electrocardiogram (EKG) showed transient accelerated idioventricular rhythm with diffuse ST depression. Initial echocardiogram on admission revealed normal left ventricular systolic function (ejection fraction (EF): 60%-65%) without valvular lesions. During her ICU stay, she underwent left internal jugular trialysis catheter insertion, and continuous hemodialysis was started for a short time. Subsequently, her creatinine normalized.

**Figure 1:**
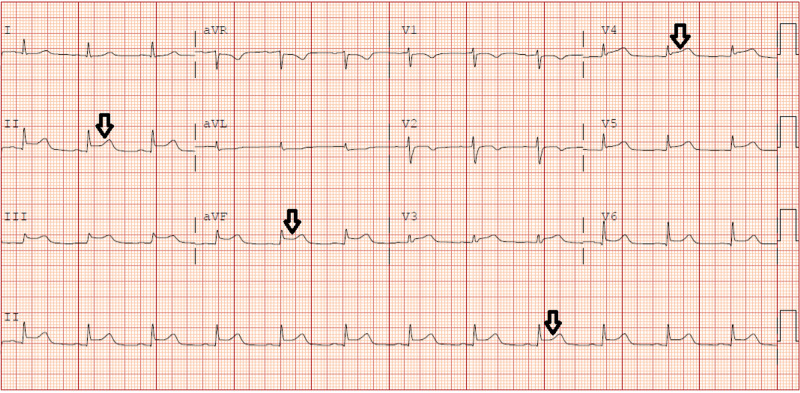
Initial electrocardiograph showing ST elevations in leads II, III, aVF, and V3-6

**Figure 2:**
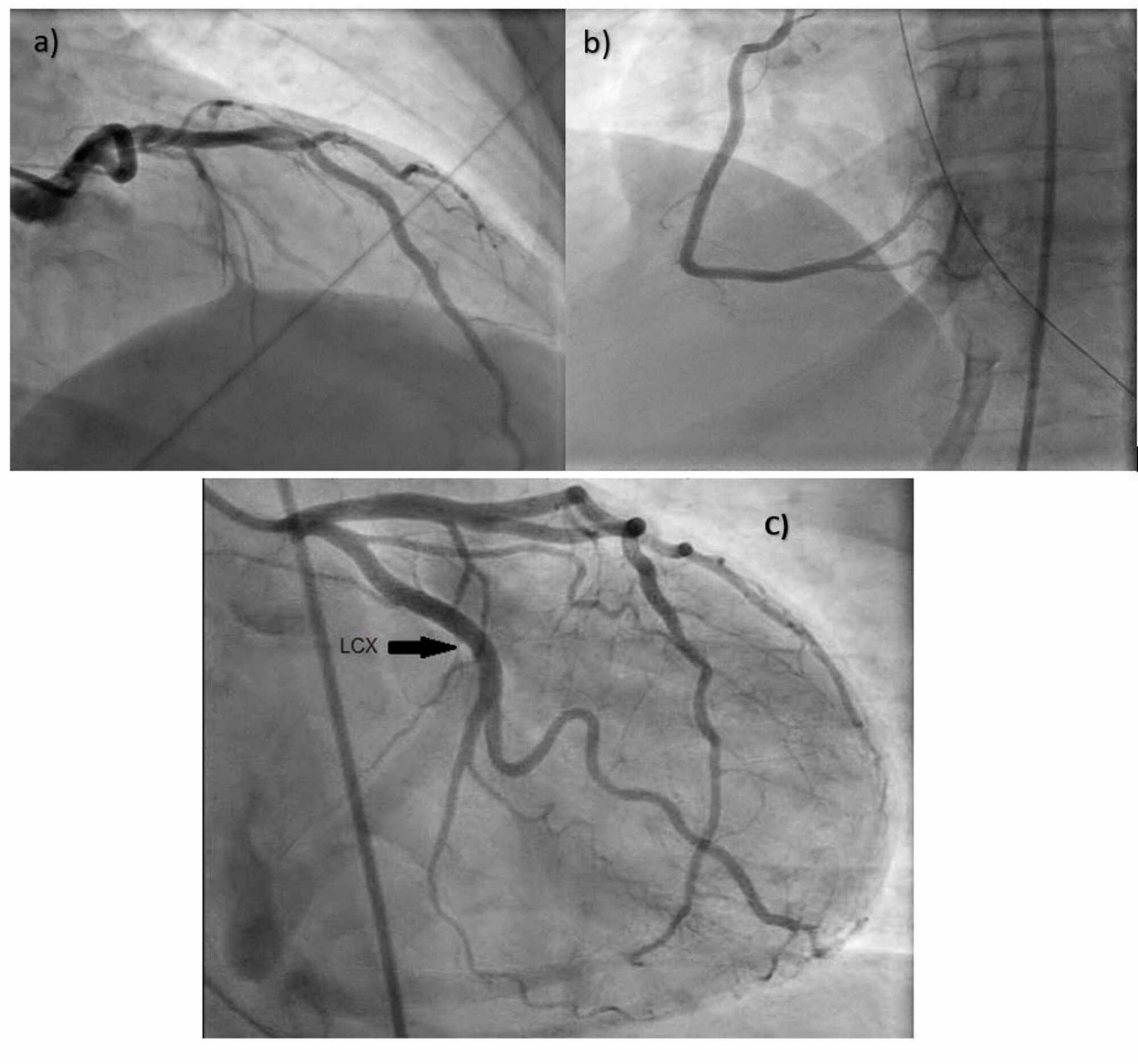
Left heart catheterization showing normal finding of left (a), right (b), and left circumflex (c) coronary arteries

**Figure 3:**
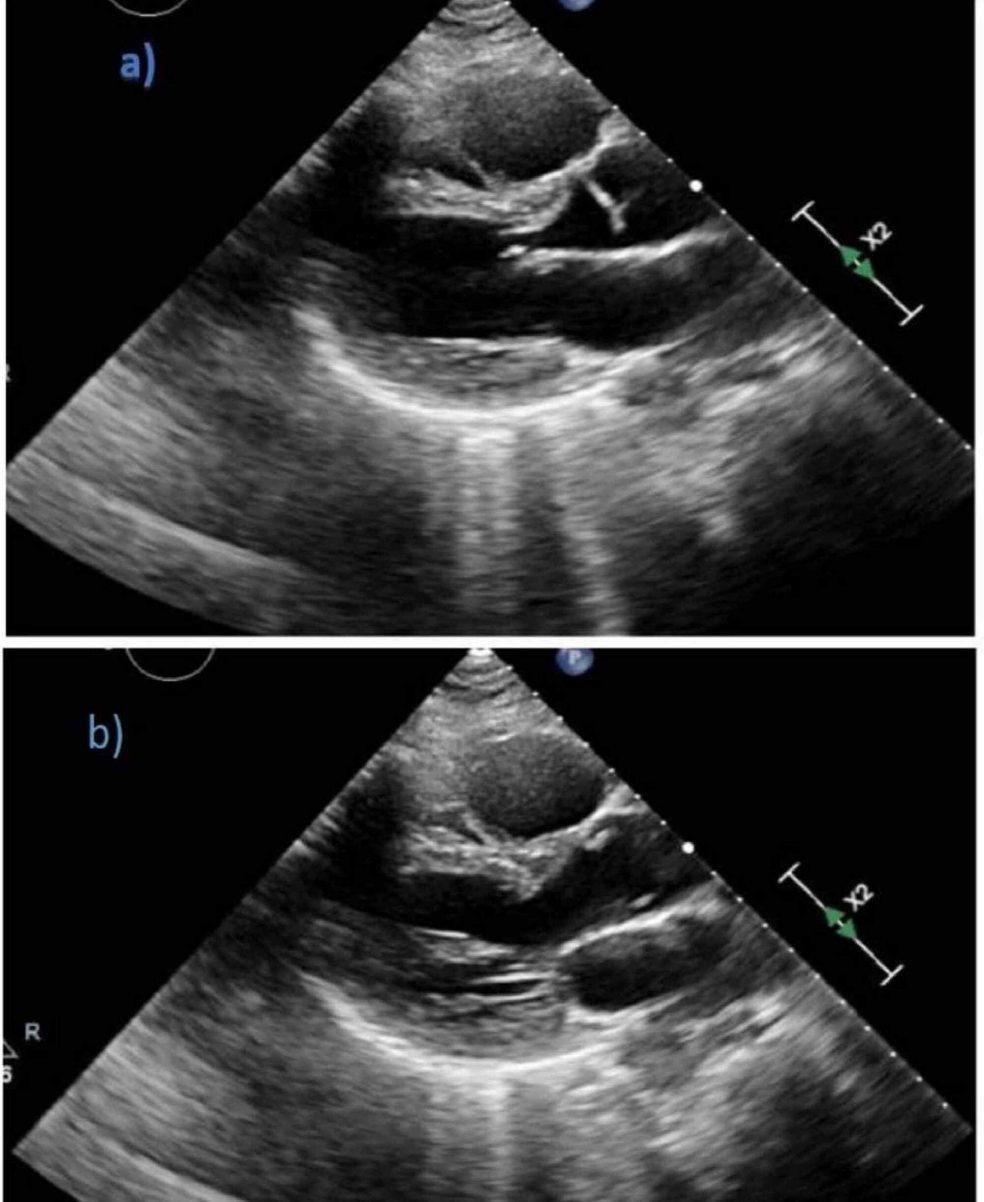
Transthoracic echocardiogram showing global hypokinesis of the left ventricle with an estimated ejection fraction (EF) of 25% during diastole (a) and systole (b) on parasternal long-axis view

On Day 2 of admission, the patient's blood pressure further dropped despite being on vasopressors. An urgent EKG revealed diffuse ST-segment elevation more in inferolateral leads (Figure 1), and her troponin levels were more than 20 ng/ml. However, emergent left heart catheterization revealed normal coronary anatomy but decreased cardiac index of 1.81 (Figure 2). Her transthoracic echocardiogram showed a globally hypokinetic left ventricle and a severely depressed ejection fraction of 25% (Figure 3). Pulmonary CT angiography was done, which ruled out pulmonary embolism. Eventually, she was diagnosed with TC. The patient was started on beta-blockers and angiotensin-converting enzyme inhibitors for her heart failure with reduced ejection fraction.

The patient was successfully extubated. Due to the decrease in EF and 16 beats run of non-sustained ventricular tachycardia, the patient was fitted for a life vest. Follow-up EKG showed no ST-segment changes, and troponin levels trended down gradually throughout the hospital stay. The patient was transferred back to an insulin pump that she uses outpatient. Her troponin was normal at 0.02 ng/ml at her one-month follow-up visit.

## Discussion

Here, we describe a case of stress-induced cardiomyopathy induced by both DKA and hypothermia.

Stress-induced cardiomyopathy or TC is a reversible condition of LV dysfunction, which presents similarly to ACS but with normal coronaries on angiogram [7-9].

TC was first reported in 1990 in Japan and has since been increasingly reported worldwide [1]. The incidence of TC among individuals exposed to physical or emotional stress is not known [10]. A prospective study showed that 28% of the cases admitted to the ICU with a non-cardiac diagnosis had apical ballooning of LV fitting TC [10]. It is estimated that the prevalence of TC is 1%-2% in patients presenting with troponin positive or ST-segment elevation suspected ACS [5,11]. TC is far more prevalent in women than in men, typically in postmenopausal Caucasian women [2].

TC is frequently but not always induced by physical and emotional stress [12]. The International Takotsubo Registry Study showed that 36% of cases were associated with a physical trigger (such as infection, central nervous system diseases, or acute respiratory failure), 27.7% of cases were associated with an emotional trigger (grief, anxiety, anger, or financial problems), and 7.8% of cases were associated with both physical and emotional triggers [12]. However, 28.5% of cases had no obvious cause [12].

The exact pathophysiology of TC is not well-understood; the most accepted postulated mechanism is a catecholamine surge [13]. DKA is an overwhelming process that physiologically increases stress hormones such as catecholamines, cortisol, glucagon, and growth hormone [14]. The cardiac apex has a high amount of beta-1 adrenergic receptors where the catecholamines hyperstimulation exerts the strongest impact resulting in myocardial stunning and hypokinetic apex often recognized as “apical ballooning” [15]. Furthermore, more ketones are produced by the liver during DKA to serve as an energy substrate for the heart [16]. When the concentration of the ketones is high enough, the patient will have acidosis, which has a deleterious effect on the myocyte [16]. The sarcoplasmic reticulum is the most affected part of the myocyte, resulting in an impairment of its ability to release calcium [16]. The ultimate effect is a stunned muscle that is unable to have a normal contraction [1,16].

Hypothermia is induced by a variety of conditions, including hypothyroidism, adrenal insufficiency, sepsis, or drug intoxication [14]. Our patient did not have any of the preceding possibilism; we assumed that the shock status as a result of DKA was responsible for poor perfusion and hypothermia.

Very few cases of TC induced by DKA have been described in the literature. The first case of DKA-induced TC reported in the literature was in 2009 by Nanda et al. [17]. Since then, more cases of TC induced by DKA were reported [17-19]. However, Katayama et al. reported the first case of TC induced by both DKA and hypothermia [14]. To our knowledge, we describe the second case of Takotsubo cardiomyopathy triggered by both DKA and hypothermia.

The clinical manifestation of TC is identical to ACS, and the most common presenting symptom is acute chest pain, but some patients present with dyspnea or syncope [12]. Other patients may develop symptoms and signs of heart failure, arrhythmias, sudden cardiac arrest, or significant mitral regurgitation [7]. Approximately 10% of patients develop symptoms and signs of cardiogenic shock (such as hypotension, abnormal mental status, cold extremities, oliguria, or respiratory distress) [12]. Cardiac catheterization is essential to exclude underlying obstructive coronary artery disease, as TC and ACS have similar presentation, elevated cardiac markers, and similar EKG changes [12].

Stress-induced cardiomyopathy can present with different patterns of ventricular wall dysfunction [5,12]. Those patterns include the apical type (which is the most common type, and atypical variants such as midventricular, basal, focal, and global types [5,12]. The global pattern of TC is fairly rare [20]. Our patient demonstrated global LV hypokinesis (Figure 3) in addition to the normal coronary angiogram, which confirms the diagnosis of stress-induced cardiomyopathy or TC (Figure 2).

The treatment of TC is typically supportive, and resolution is seen within two months. Every effort should be made to control the underlying cause; the goal is to treat the triggering cause first [16]. In our case, when the DKA was treated and acidosis resolved, repeated EKG showed improvement in the ejection fraction and normal ventricular motion, therefore, the overall resolution of the TC.

Physicians should always suspect TC in any patient with heart failure during DKA treatment after ruling out other causes, such as ACS and valvular heart diseases, since the treatment and prognosis are different. Reduced EF seen in TC can make fluid resuscitation in DKA challenging as well.

## Conclusions

Both hypothermia and DKA can trigger TC. Catecholamine excess is thought to be the culprit in the mechanism of TC. Physicians should always consider TC in any patient with heart failure during DKA treatment after the exclusion of other causes, including ACS and valvular heart diseases. Although TC is a transient condition, it is associated with dreadful complications, including arrhythmias, cardiogenic shock, or thromboembolic events. Early recognition and timely treatment are pivotal in such cases.

## References

[1] Akashi YJ, Nef HM, Lyon AR (2015). Epidemiology and pathophysiology of Takotsubo syndrome. Nat Rev Cardiol.

[2] Merchant EE, Johnson SW, Nguyen P, Kang C, Mallon WK (2008). Takotsubo cardiomyopathy: a case series and review of the literature. West J Emerg Med.

[3] Medina de Chazal H, Del Buono MG, Keyser-Marcus L, Ma L, Moeller FG, Berrocal D, Abbate A (2018). Stress cardiomyopathy diagnosis and treatment: JACC state-of-the-art review. J Am Coll Cardiol.

[4] Gianni M, Dentali F, Grandi AM, Sumner G, Hiralal R, Lonn E (2006). Apical ballooning syndrome or takotsubo cardiomyopathy: a systematic review. Eur Heart J.

[5] Kurowski V, Kaiser A, von Hof K (2007). Apical and midventricular transient left ventricular dysfunction syndrome (tako-tsubo cardiomyopathy): frequency, mechanisms, and prognosis. Chest.

[6] Sheppard MN (2015). Takotsubo syndrome - stress-induced heart failure syndrome. Eur Cardiol.

[7] Bybee KA, Kara T, Prasad A, Lerman A, Barsness GW, Wright RS, Rihal CS (2004). Systematic review: transient left ventricular apical ballooning: a syndrome that mimics ST-segment elevation myocardial infarction. Ann Intern Med.

[8] Tsuchihashi K, Ueshima K, Uchida T (2001). Transient left ventricular apical ballooning without coronary artery stenosis: a novel heart syndrome mimicking acute myocardial infarction. J Am Coll Cardiol.

[9] Abe Y, Kondo M, Matsuoka R, Araki M, Dohyama K, Tanio H (2003). Assessment of clinical features in transient left ventricular apical ballooning. J Am Coll Cardiol.

[10] Park JH, Kang SJ, Song JK, Kim HK, Lim CM, Kang DH, Koh Y (2005). Left ventricular apical ballooning due to severe physical stress in patients admitted to the medical ICU. Chest.

[11] Prasad A, Dangas G, Srinivasan M, Yu J, Gersh BJ, Mehran R, Stone GW (2014). Incidence and angiographic characteristics of patients with apical ballooning syndrome (Takotsubo/stress cardiomyopathy) in the HORIZONS-AMI trial: an analysis from a multicenter, international study of ST-elevation myocardial infarction. Catheter Cardiovasc Interv.

[12] Templin C, Ghadri JR, Diekmann J (2015). Clinical features and outcomes of Takotsubo (stress) cardiomyopathy. N Engl J Med.

[13] Paur H, Wright PT, Sikkel MB (2012). High levels of circulating epinephrine trigger apical cardiodepression in a beta2-adrenergic receptor/Gi-dependent manner. A new model of Takotsubo cardiomyopathy. Circulation.

[14] Katayama Y, Hifumi T, Inoue J, Koido Y (2013). A case of Takotsubo cardiomyopathy induced by accidental hypothermia and diabetic ketoacidosis. BMJ Case Rep.

[15] Akashi YJ, Nakazawa K, Sakakibara M, Miyake F, Musha H, Sasaka K (2004). 123I-MIBG myocardial scintigraphy in patients with "Takotsubo" cardiomyopathy. J Nucl Med.

[16] Gordon A, LaCapra G, Roberti R (2017). DKA-induced Takotsubo cardiomyopathy in patient with known HOCM. Case Rep Crit Care.

[17] Nanda S, Longo S, Bhatt SP, Pamula J, Sharma SG, Dale TH (2009). Stress cardiomyopathy - a unique presentation of diabetic ketoacidosis. Ann Clin Biochem.

[18] Meyers JH, Hirsch IB (2017). Takotsubo cardiomyopathy in association with DKA in a blind pump patient. AACE Clin Case Rep.

[19] Khan M, Khalid S, Marwat A, Mehmood H (2018). A case of euglycemic diabetic ketoacidosis due to canagliflozin complicated by Takotsubo cardiomyopathy. Am J Med Case Rep.

[20] Win CM, Pathak A, Guglin M (2011). Not Takotsubo: a different form of stress-induced cardiomyopathy—a case series. Congest Heart Fail.

